# Whole-brain irradiation with hippocampal sparing and dose escalation on metastases: neurocognitive testing and biological imaging (HIPPORAD) – a phase II prospective randomized multicenter trial (NOA-14, ARO 2015–3, DKTK-ROG)

**DOI:** 10.1186/s12885-020-07011-z

**Published:** 2020-06-08

**Authors:** Anca-Ligia Grosu, Lars Frings, Iryna Bentsalo, Oliver Oehlke, Franziska Brenner, Angelika Bilger, Jamina Tara Fennell, Thomas Rothe, Sabine Schneider-Fuchs, Erika Graf, Claudia Schmoor, Jürgen Beck, Gerhild Becker, Michael Bock, Karl Egger, Horst Urbach, Claas Lahmann, Ilinca Popp

**Affiliations:** 1 Department of Radiation Oncology, Faculty of Medicine, Medical Center – University of Freiburg, Robert-Koch-Str. 3, 79106 Freiburg, Germany; 2 German Cancer Consortium (DKTK), Partner Site Freiburg, German Cancer Research Center (DKFZ), Heidelberg, Germany; 3 Present affiliation: Department of Nuclear Medicine, Faculty of Medicine, Medical Center-University of Freiburg, Freiburg, Germany; 4 Department of Psychosomatic Medicine and Psychotherapy, Faculty of Medicine, Medical Center – University of Freiburg, Hauptstraße 8, 79104 Freiburg, Germany; 5 Present affiliation: Department of Radiation Oncology, Kliniken Maria Hilf GmbH Mönchengladbach, Mönchengladbach, Germany; 6 Present affiliation: Department of Radiation Oncology, Ortenau-Klinikum Offenburg-Gengenbach, Offenburg, Germany; 7 Clinical Trials Unit, Faculty of Medicine, Medical Center – University of Freiburg, Elsässer Straße 2, 79110 Freiburg, Germany; 8 Institute of Medical Biometry and Statistics, Faculty of Medicine, Medical Center – University of Freiburg, Stefan-Meier-Str. 26, 79104 Freiburg, Germany; 9 Department of Neurosurgery, Faculty of Medicine, Medical Center – University of Freiburg, Breisacher Str. 64, 79106 Freiburg, Germany; 10 Department of Palliative Care, Faculty of Medicine, Medical Center - University of Freiburg, Robert-Koch-Str. 3, 79106 Freiburg, Germany; 11 Medical Physics, Department of Radiology, Faculty of Medicine, Medical Center - University of Freiburg, Killian Str. 5a, 79106 Freiburg, Germany; 12 Department of Neuroradiology, Faculty of Medicine, Medical Center – University of Freiburg, Breisacher Straße 64, 79106 Freiburg, Germany

**Keywords:** Brain metastases, Hippocampus avoidance, Whole brain radiation therapy, Neurocognitive function

## Abstract

**Background:**

Whole brain radiation therapy (WBRT) is the standard therapy for multiple brain metastases. However, WBRT has a poor local tumor control and is associated with a decline in neurocognitive function (NCF). Aim of this trial is to assess the efficacy and safety of a new treatment method, the WBRT with hippocampus avoidance (HA) combined with the simultaneous integrated boost (SIB) on metastases/resection cavities (HA-WBRT+SIB).

**Methods:**

This is a prospective, randomized, two-arm phase II multicenter trial comparing the impact of HA on NCF after HA-WBRT+SIB versus WBRT+SIB in patients with multiple brain metastases. The study design is double-blinded. One hundred thirty two patients are to be randomized with a 1:1 allocation ratio. Patients between 18 and 80 years old are recruited, with at least 4 brain metastases of solid tumors and at least one, but not exceeding 10 metastases ≥5 mm. Patients must be in good physical condition and have no metastases/resection cavities in or within 7 mm of the hippocampus. Patients with dementia, meningeal disease, cerebral lymphomas, germ cell tumors, or small cell carcinomas are excluded. Previous irradiation and resection of metastases, as well as the number and size of metastases to be boosted have to comply with certain restrictions. Patients are randomized between the two treatment arms: HA-WBRT+SIB and WBRT+SIB. WBRT is to be performed with 30 Gy in 12 daily fractions and the SIB with 51 Gy/42 Gy in 12 daily fractions on 95% of volume for metastases/resection cavities. In the experimental arm, the dose to the hippocampi is restricted to 9 Gy in 98% of the volume and 17Gy in 2% of the volume. NCF testing is scheduled before WBRT, after 3 (primary endpoint), 9, 18 months and yearly thereafter. Clinical and imaging follow-ups are performed 6 and 12 weeks after WBRT, after 3, 9, 18 months and yearly thereafter.

**Discussion:**

This is a protocol of a randomized phase II trial designed to test a new strategy of WBRT for preventing cognitive decline and increasing tumor control in patients with multiple brain metastases.

**Trial registration:**

The HIPPORAD trial is registered with the German Clinical Trials Registry (DRKS00004598, registered 2 June 2016).

## Background

Brain metastases are the most common intracranial tumors in adults, occurring in 10–30% of patients with systemic malignancies [1]. For patients with multiple brain metastases, whole brain radiation therapy (WBRT) remains the primary treatment modality [2].

WBRT was shown to significantly improve distant intracerebral tumor control and reduce the rate of neurological death compared to radiosurgery alone [3], leading to the assumption of relevant microscopic tumor infiltration of the brain. Consequently, the whole brain can be considered a clinical target volume (CTV) and would benefit from a moderate radiation dose [3].

However, WBRT is associated with long-term side effects. Patients receiving additional WBRT after local therapy reported significantly poorer quality of life (QoL) scores than patients receiving local therapy only [4]. The differences were clinically relevant as early as 8 weeks after treatment. The main culprit are neurocognitive deficits frequently observed after WBRT of multiple brain metastases, with episodic memory being the predominant cognitive domain affected [5–7]. Memory deficits are correlated with and precede decreased quality of life [8], and thus play an essential role in patients’ subjective well-being. Although other factors contribute to memory decline, such as age, tumor growth or medication, WBRT has clearly been demonstrated to significantly impair memory [5, 9].

The hippocampus is the human brain structure that is most critically involved in episodic memory processing [10, 11]. Damage to the hippocampi can lead to profound amnestic syndromes [12]. It has been demonstrated that a reduction of adult neurogenesis within the hippocampus leads to hippocampal dysfunction [13, 14] and that irradiation blocks adult neurogenesis in the subgranular zone of the hippocampus [15]. Episodic memory deficits after WBRT have therefore been concluded to be associated with hippocampal damage [16, 17]. Hippocampal damage might be prevented by hippocampus avoidance-WBRT (HA-WBRT), which is hypothesized to attenuate cognitive decline as measured by standardized neuropsychological testing. Results of the RTOG 0933, a single-arm non-randomized phase II trial of conformal HA during WBRT, showed a preservation of memory and QoL compared to a historical series of patients receiving standard WBRT [18]. The NRG Oncology CC001 phase III randomized trial investigating WBRT and memantine hydrochloride versus HA-WBRT and memantine hydrochloride recently showed a significantly higher risk of neurocognitive failure in the conventional WBRT arm [19]. The avoidance of the hippocampus during WBRT is considered to be a safe treatment concept, with a rate of (peri)hippocampal disease progression after HA-WBRT according to previous data between 7.6 and 12.1% [20–22].

Apart from the HA-WBRT, the current trial plans an additional boost to the metastases with the aim of obtaining an improved local tumor control (LTC). Progressive brain metastases have been correlated with significant neurocognitive deterioration [23–25], suggesting that LTC can have an essential contribution to the preservation of cognitive functions. In our pilot cohort, the LTC of boosted metastases and the intracranial progression-free survival (PFS) were significantly higher after HA-WBRT+SIB compared to conventional WBRT, emphasizing the potential benefit of intensified local treatment even for patients with more than 4 brain metastases [22]. The dose escalation in the form of sequential radiosurgeries after WBRT was also previously investigated by Andrews et al. and showed improved LTC and overall survival (OS) rates [26].

In the preparation of this clinical trial protocol, a planning study was performed, in search for the best state-of-the-art radiotherapy technique to safely apply the required dose, while providing optimal HA [27]. Both a simultaneous integrated boost (SIB) and a sequential approach were investigated and both were able to achieve adequate whole brain coverage and radiosurgery-equivalent dose distributions to individual brain metastases. The SIB technique however achieved better sparing of the hippocampi, especially when a hippocampal avoidance region > 5 mm was used. Consequently, this approach is used in the context of this trial.

Thus, the current project aims to prospectively investigate the benefits of the novel, recently established technique of HA-WBRT+SIB in comparison to WBRT+SIB without HA on the memory performance of patients treated for brain metastases. The main hypothesis of this study is that HA-WBRT+SIB minimizes the side effect of cognitive deterioration and shows similar efficacy with regard to LTC and survival as compared to WBRT+SIB. Moreover, the study will assess safety, as well as structural and functional changes of the brain using magnetic resonance imaging (MRI).

## Methods

### Trial design and setting

This is a prospective, randomized, two-arm phase II multicenter trial comparing the impact of hippocampal sparing on the neurocognitive function (NCF) during HA-WBRT+SIB versus WBRT+SIB in patients with at least 4 brain metastases. The study design is double-blinded, with patients and assessors of NCF and MRI blinded to the assigned treatment arm. One hundred thirty two patients are to be randomized with a 1:1 allocation ratio to the treatment arms. Due to the anticipated loss during the screening period, we expect that 160 patients will have to be screened for enrolment.

Trial sites are academic hospitals and community clinics located in Germany and Switzerland: Bad Saarow, Dresden (two sites), Dessau, Freiburg, Frankfurt (two sites), Hamburg, Hameln, Kiel, Cologne, Moenchengladbach, Munich (two sites), Regensburg, Wolfsburg, Wuerzburg, Tuebingen, Zurich (two sites).

The trial and its amendments (current version: V.3.0 / 27.08.2018) were approved by the ethics committee of the University of Freiburg (EK-Freiburg 108/16) and by the local ethics committees of participating sites. The trial is associated with the German Cancer Society Neuro-Oncology Working Group NOA (Neuroonkologische Arbeitsgemeinschaft, NOA-14), the Radiation Oncology Group (Arbeitsgemeinschaft Radiologische Onkologie, ARO 2015–3) of the German Cancer Society (Deutsche Krebsgesellschaft, DKG) and the Radiation Oncology Group of the German Cancer Consortium (DKTK-ROG) and is funded by the German Cancer Aid (Deutsche Krebshilfe, Grant No. 110912).

### Study population

The target population for this trial consists in patients with at least 4 brain metastases of solid tumors, with at least one, but not exceeding 10 metastases ≥5 mm (i.e., eligible for dose escalation). No gender ratio has been stipulated in this study as the results of the preclinical and/or clinical studies did not indicate any difference in the effect of the study treatment in terms of efficacy and safety. However, patients over the age of 80 are excluded, as NCF tests have not been validated for older age cohorts.

Inclusion criteria to be met at the time of registration/randomisation are:

1. Patient’s written informed consent obtained,
2. Age 18–80 years, male or female,
3. Legal capacity, patient is able to understand the nature, significance, and consequences of the trial,
4. At least 4 brain metastases of solid tumors, with at least one, but not exceeding 10 metastases ≥5 mm (i.e., eligible for dose escalation),
5. Recursive partitioning analysis classification I or II,
6. No metastases (either pre-irradiated or not) and no resection cavity within the hippocampus or in a distance of 7 mm to the hippocampus (= hippocampal avoidance region, HAR).

Exclusion criteria are:

1. Simultaneous participation in other interventional trials which could interfere with this trial,
2. Participation in a clinical trial within the last thirty days before the start of this trial, previous participation (randomization) in this trial,
3. Known or persistent abuse of medication, drugs or alcohol,
4. Persons who are in a relationship of dependence/employment with the sponsor or the investigator,
5. Pregnancy, nursing or patient not willing to prevent a pregnancy during treatment,
6. Cerebral lymphomas, metastases of germ cell tumors, small cell lung cancer,
7. Acute neurological symptoms demanding an immediate start of radiation therapy,
8. Central nervous system diseases or syndromes accompanied by cognitive deficits or radiological changes of the brain, e.g., dementia (VLMT-Learning < 39; VLMT-Delayed Recall < 7), major depression, clinically manifest hypertensive encephalopathy, carcinomatous meningitis,
9. Previous brain irradiation (radiosurgery/stereotactic fractionated radiotherapy (SFRT)) of > 1 brain metastasis > 3 cm or > 3 brain metastases > 1 cm each,
10. Previous brain irradiation < 3 months before start of treatment,
11. Previous surgical resection (± adjuvant SFRT) of > 1 brain metastasis (biopsy allowed),
12. Previous surgical resection of 1 brain metastasis < 4 weeks before start of treatment,
13. Uncontrolled pretreated brain metastasis/−es after radiosurgery/SFRT,
14. Last application of chemotherapy/ immunotherapy/ targeted therapy < 1 week before randomization,
15. Radiotherapy planning conforming to organ at risk (OAR) constraints not feasible (including previous cranial irradiation),
16. Benzodiazepines, barbiturates, topiramate, hydantoine as antiepileptic medication,
17. More than 1 brainstem metastasis ≥5 mm,
18. Brainstem metastasis > 2 cm,
19. Brain metastasis > 3.5 cm,
20. Resection cavity eligible for dose escalation > 3.5 cm.

### Study treatment and procedures

Patients eligible and willing to participate are randomized to one of two treatment arms – HA-WBRT+SIB vs. WBRT+SIB. The schedule of enrolment, interventions and assessments are provided in Table 1. Each study center is asked to participate in a radiotherapy-planning dummy run before the inclusion of the first trial patient in order to ensure uniformity in image co-registration, contouring and treatment planning.

**Table 1:** Flowchart of enrolment, interventions and assessments

	Staging	Therapy		Follow-up				
	T_**0**_	Start	End	T_**1**_	T_**2**_	T_**3**_	T_**4**_	T_**5**_ – T_**x**_
	1 week before RT (day − 6 until day 0)	Day 1 of RT	Day 12 of RT	6 weeks after RT (+/− 7 days)	3 months after RT (+/− 10 days)	9 months after RT (+/− 10 days)	18 months after RT (+/− 10 days)	every 12 months, starting at 30 months after RT (+/− 28 days)
Check of in−/ exclusion criteria	**X**							
Patient information, informed consent	**X**							
Registration	**X**							
Randomization	**X**							
**TREATMENT PLANNING AND IMAGE ACQUISITION**								
Planning of Radiotherapy	**X**							
Upload of planning CT and structure sets	**X**							
Upload of MR images	**X**			**X**	**X**	**X**	**X**	**X**
**DOCUMENTATION**								
Documentation of demographic data and medical history	**X**							
Clinical, neurological examination	**X**	**X**		**X**	**X**	**X**	**X**	**X**
Documentation of medication	**X**	**X**		**X**	**X**	**X**	**X**	**X**
Documentation of adverse events		**X**		**X**	**X**	**X**	**X**	**X**
**ASSESSMENT OF TARGET VARIABLES**								
NCF Assessment (VLMT, WMS-R-Digit Spans, TMT-A /−B)	**X**				**X**	**X**	**X**	**X**
Depression Assessment (HADS-D)	**X**				**X**	**X**	**X**	**X**
QoL Assessment (QLQ-C15-PAL/−BN20, SEIQoL-Q)	**X**				**X**	**X**	**X**	**X**
Study-specific MRI	**X**			**X**	**X**	**X**	**X**	

All patients undergo a planning computed tomography (CT) with 1–2 mm slice thickness in mask immobilization, as well as MRI imaging using a minimum 1.5 Tesla MR-scanner. Minimum MRI requirement is the acquisition of a three dimensional (3D) T1-weighted sequence with 1 mm^3^ large isotropic voxels before and after intravenous application of a Gadolinium-containing contrast medium. The MRI datasets (Gd-T1-MRI) are co-registered on the planning-CT acquired in treatment position. Non-elastic registration has to be performed; non-rigid methods are not allowed. The quality of co-registration is to be checked by the radiation oncologist before target volume contouring by visual inspection of anatomical landmarks in all image modalities for topographical matching.

**Fig. 1:**
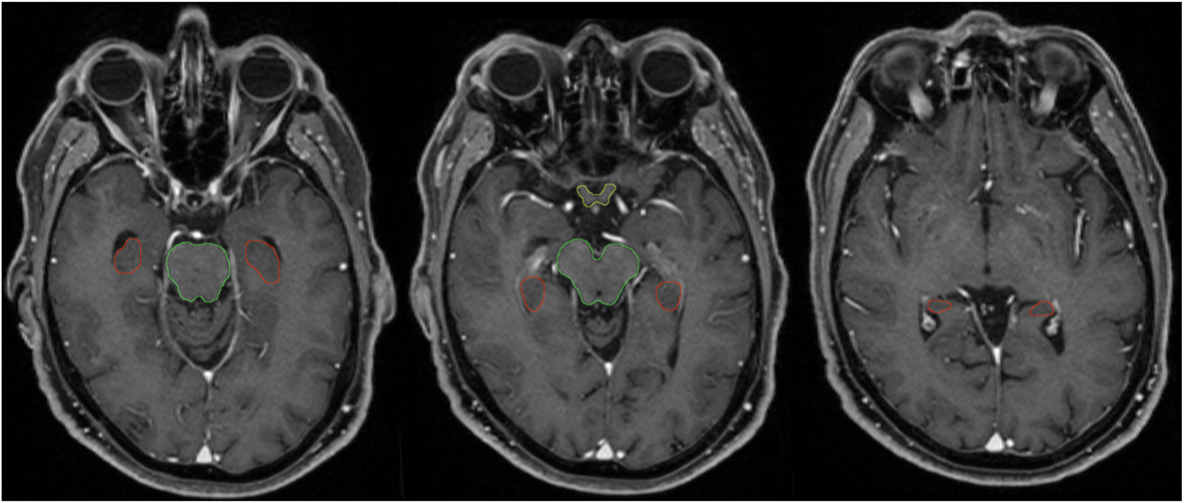
Hippocampus contouring (red) according to the contouring atlas for RTOG 0933 [18]

Gross tumor volume for each metastasis (GTV_met_) is defined as the contrast-enhancing lesion on Gd-T1-MRI. Planning tumor volume for metastases, PTV_met_ is produced as 1 mm 3D margin expansion of the GTV_met_. CTV for whole brain (CTV_WB_) is defined as the whole-brain parenchyma. Planning target volume (PTV) for whole brain in Arm A, PTV_WB-A_, is defined as the CTV_WB_ + 5 mm excluding the PTV_met_ and the hippocampal avoidance regions (HAR). Planning target volume for whole brain in Arm B, PTV_WB-B_, is defined as the CTV_WB_ + 5 mm excluding the PTV_met_. Planning target volume (PTV) for resection cavity, PTV_res_, is defined as the CTV_res_, including the potential GTV of contrast enhancement areas around the resection cavity (tumor rests), + 2 mm. Bilateral hippocampal contours are manually generated on the fused MRI and CT planning image set by physicians according to the contouring atlas for RTOG 0933 (Fig. 1) [18]. HAR is defined as a 7 mm 3D expansion around the hippocampus.

**Fig. 2:**
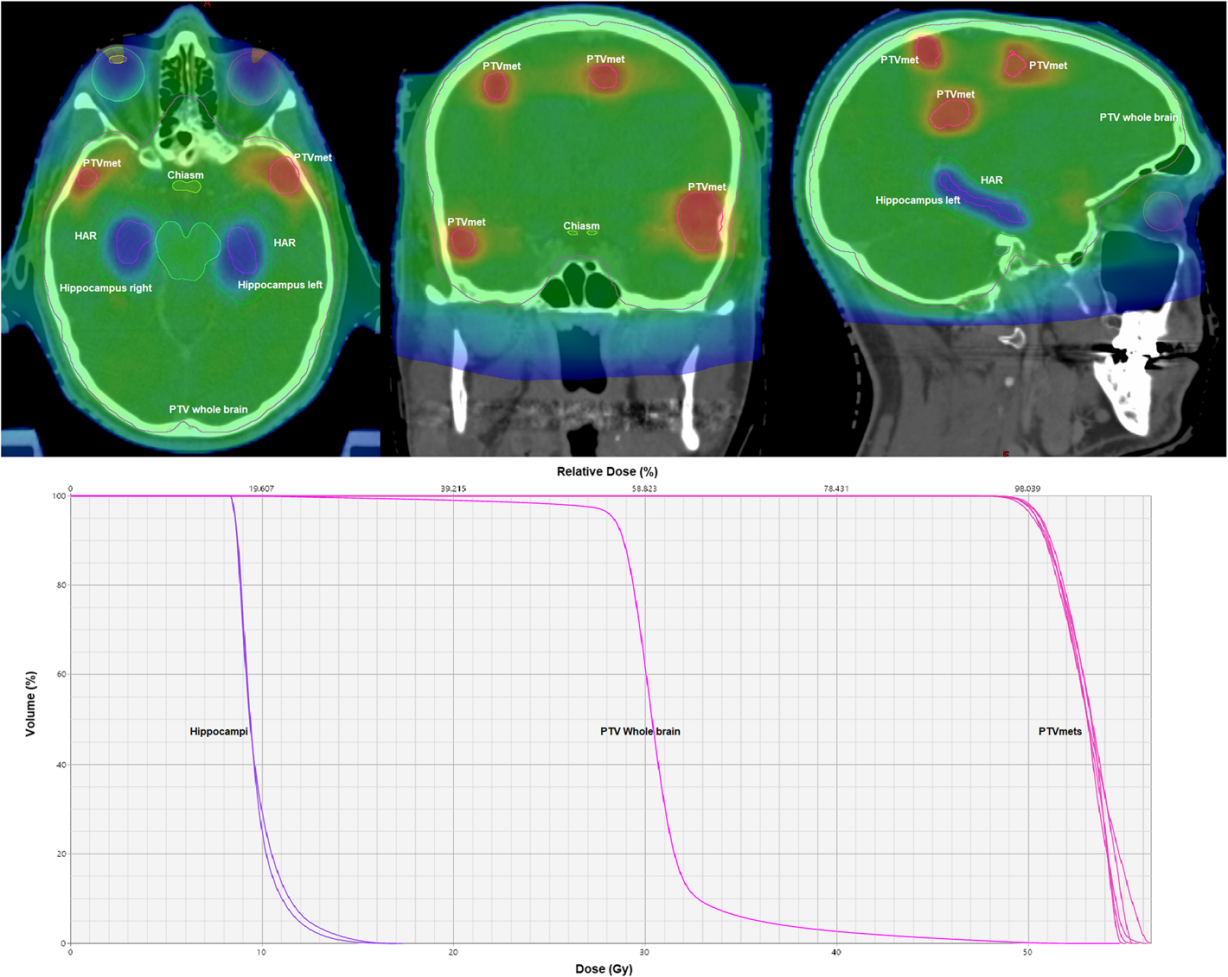
Example of dose distribution and dose-volume histogram of a HA-WBRT+SIB plan in a patient with 7 brain metastases. HAR = hippocampus avoidance region; PTVmet = planning target volume for metastasis (gross tumor volume + 1 mm); PTV Whole brain (whole brain + 5 mm – HAR– PTVmets). Mean dose in the hippocampi under 10 Gy, mean dose for whole brain and metastases 30 and 51 Gy respectively

Linear accelerator-based intensity-modulated radiation therapy and volumetric modulated arc therapy techniques or helical tomotherapy are required for planning (Fig. 2).

The prescribed dose is 30Gy in 12 fractions for PTV_WB-A_ or PTV_WB-B_ and 51Gy in 12 fractions (4.25Gy/fraction on 95% of volume) as SIB to individual brain metastases (brain stem metastasis and resection cavities: 42Gy in 12 fractions, 3.5Gy/fraction). In the experimental arm, a simultaneous integrated protection (SIP) to the hippocampi is applied, with a dose in 98% of the volume (D_98%_) ≤ 9Gy and in 2% of the volume (D_2%_) ≤ 17Gy [28]. In the control arm, D_98%_ to the hippocampi must be kept ≥25Gy. A high precision conformal radiotherapy with a steep dose gradient is required. Treatment is to be delivered once daily, with 5 fractions per week. Detailed information on the applied dose and on constraints for organs at risk can be seen in Tables 2, 3, 4.

**Table 2:** Treatment planning (PTV_WB-A_ = Planning target volume of the whole brain in arm A, PTV_WB-B_ = Planning target volume of the whole brain in arm B, PTV_met_ = Planning target volume of metastases)

Radiation treatment planning	PTV_**WB- A or -B**_	PTV_**met**_	Major deviations
Prescribed total dose to PTV	30 Gy	51Gy (brain stem metastasis and resection cavity: 42Gy)	< or > 30Gy for PTV_WB-A or -B_ < or > 51Gy for PTV_met_ (brain stem metastasis and resection cavity: < or > 42Gy)
Prescribed dose per fraction in PTV	2.5 Gy	4.25Gy (brain stem metastasis and resection cavity: 3.5Gy)	< or > 2.5Gy for PTV_WB-A or -B_ < or > 4.25Gy for PTV_met_ (brain stem metastasis and resection cavity: 3.5Gy)
Number of fractions per day	1	1	> 1
Treatment duration	12 days 5 days/week	12 days 5 days/week	< or > 12 days < 4 or > 5 days/week

**Table 3:** Planning target volume (PTV) – specifications for total dose (PTV_WB-A_ = Planning target volume of the whole brain in arm A, PTV_WB-B_ = Planning target volume of the whole brain in arm B, PTV_met_ = Planning target volume of metastases, PTV_res_ = Planning target volume of resection cavity)

Parameter	Per Protocol	Minor Deviation	Major Deviation
PTV_WB-A or -B_	D_98%_ ≥ 25Gy D_mean_ ≤ 35 Gy	24Gy ≤ D_98%_ < 25Gy 35Gy < D_mean_ ≤ 37 Gy	D_98%_ < 24Gy D_mean_ > 37 Gy
PTV_met 51Gy (brain metastases)_	D_98%_ ≥ 48.4Gy D_2%_ ≤ 63.7Gy	47.4Gy ≤ D_98%_ < 48.4Gy 63.7Gy < D_2%_ ≤ 66.3Gy	D_98%_ < 47.4Gy D_2%_ > 66.3Gy
PTV_met 42Gy_ (brain stem metastases) PTV_res 42Gy_	D_98%_ ≥ 39.9Gy D_2%_ ≤ 52.5 Gy	39.06 Gy ≤ D_98%_ < 39.9Gy 52.5 Gy < D_2%_ ≤ 54.6 Gy	D_98%_ < 39.06 D_2%_ > 54.6 Gy

*PTV_*WB-A*_

**Table 4:** Organs at risk (OAR)

Parameter	Per Protocol	Minor Deviation	Major Deviation
Hippocampus in Treatment Arm A (HA-WBRT+SIB)	D_98%_ ≤ 9Gy D_2%_ ≤ 17Gy	9 Gy < D_98%_ ≤ 10Gy 17Gy < D_2%_ ≤ 18Gy	D_98%_ > 10Gy D_2%_ > 18Gy
Hippocampus in Treatment Arm B (WBRT+SIB)	D_98%_ ≥ 25Gy	24Gy ≤ D_98%_ < 25Gy	D_98%_ < 24Gy
Optical nerves Chiasm	D_2%_ ≤ 33Gy	33Gy < D_2%_ ≤ 35Gy (36Gy^a^)	D_2%_ > 35Gy (36Gy^a^)
Optical nerve, one-sided	D_2%_ ≤ 35Gy	35Gy < D_2%_ ≤ 37Gy	D_2%_ > 37Gy
Retina, one-sided	D_2%_ ≤ 33Gy	33Gy < D_2%_ ≤ 35Gy (49Gy^a^)	D_2%_ > 35Gy (49Gy^a^)
Lenses	D_2%_ ≤ 7Gy	7Gy < D_2%_ ≤ 10Gy	D_2%_ > 10Gy
Inner ear (in case of bilateral involvement)	D_2%_ ≤ 33Gy	33Gy < D_2%_ ≤ 35Gy (36Gy^a^)	D_2%_ > 35Gy (36Gy^a^)
Eyes	D_2%_ ≤ 33Gy	33Gy < D_2%_ ≤ 35Gy	D_2%_ > 35Gy
Brain stem (if metastasis inside the brainstem: brain stem-PTV_met_)	D_2%_ ≤ 33Gy	33Gy < D_2%_ ≤ 35Gy (51Gy^a^)	D_2%_ > 35Gy (51Gy^a^)

^a^in case of brain metastasis in the vicinity of OAR

Investigators enter pseudonymized patient data into electronic case report forms with automatic validation programs checking for completeness, consistency and plausibility. Randomization is performed centrally at the Medical Center – University of Freiburg and stratified by center, in blocks of variable length, based on computer-generated lists. The block lengths are documented separately and are not to be disclosed to the centers. Patients and assessors of NCF and MRI are blinded to treatment assignment. The use of different access rights to the electronic case report forms for treating radiation oncologists and for assessors of NCF and MRI assist in maintaining blinding.

During study treatment, any medication clinically indicated may be applied, with the exceptions of benzodiazepines, barbiturates, topiramate, and hydantoines used as antiepileptic medication. However, in case of an emergency, any medical treatment is allowed. Patients are to be excluded from the study in case of adverse events not allowing continuation of radiotherapy, non-hematological Common Terminology Criteria for Adverse Events (CTCAE) grade 4 toxicity, lack of cooperation, compliance or adherence to protocol, investigator judging the patient risk-benefit ratio to be unfavorable, patient taking drugs not permitted or participating in other trials, and pregnancy. Patients who withdraw from radiotherapy prematurely should still be subject to the same assessments as they would have had if they had completed the study treatment.

### Neurocognitive function assessment

The primary endpoint is the hippocampal-dependent episodic memory after treatment. Differential worsening of the two treatment groups from pre- to post-treatment is to be evaluated. The specific hypothesis to be tested is that 3 months after treatment – compared to pre-treatment – episodic memory decline in the HA-WBRT+SIB group is less severe than in the group of patients who received WBRT+SIB.

The primary endpoint will be assessed with the German version of the Auditory Verbal Learning Test [29], the VLMT (*Verbaler Lern- und Merkfähigkeitstest*) [30]. The VLMT is a standardized test with proven sensitivity to hippocampal dysfunction [31] and association with memory-related hippocampal activation [32]. Feasibility and adequacy of using the VLMT in similar settings have been demonstrated. Comparing two groups of patients with temporal lobe resections either including or sparing the hippocampus, Wagner et al. found that the patients whose hippocampus had been resected showed a significantly stronger decline of verbal learning performance after surgery as assessed with the VLMT [33]. The VLMT has also been used before in studies investigating the effects of WBRT for brain metastases [6] and chemotherapy for central nervous system lymphoma [34].

Among the secondary endpoints, episodic verbal memory and changes in delayed recall are assessed with the VLMT at additional time points: 9 and 18 months after treatment, in order to investigate acute and long-term effects of WBRT and the benefits of HA. Beyond 18 months after the end of WBRT, all patients who are still alive receive NCF assessment with VLMT once per year.

Episodic memory is a higher cognitive function that depends on basal cognitive processes like perception and attention and is also impaired by decreased information processing speed. Therefore, in the current study, short term and working memory, as well as information processing speed and cognitive flexibility are tested. Verbal short term and working memory performance are assessed with the subtests ‘Digit Span Forward’ and ‘Digit Span Backward’ from the German version of the WMS-R (Wechsler Memory Scale-Revised) [35], while information processing speed, visuo-motor tracking abilities as well as executive functions with the TMT (Trail Making Test) [36]. The ‘Digit Spans’ subtest and the TMT are applied among the secondary endpoints before and 3, 9, and 18 months after treatment. Beyond 18 months after the end of WBRT, all patients who are still alive are tested once per year.

NCF assessments are performed or supervised by experienced neuropsychologists in order to ensure standardization and comparability of results. A good compliance of patients treated for brain metastases has been demonstrated in a similar setting, even with a more extensive test battery [23].

### Assessment of depression

Depression can have a negative impact on memory performance [37, 38] and is frequent in patients treated with radiotherapy [39]. In order to account for effects of depression on the primary outcome variable, the German version of the Hospital Anxiety and Depression Scale (HADS-D) [40] is used. The baseline and depression score at 3 months are to be used for adjustment in a sensitivity analysis of the treatment effect on the primary endpoint memory performance.

Evaluation of depression is performed before, as well as 3, 9, and 18 months after treatment. Beyond 18 months after the end of WBRT, all patients who are still alive receive evaluation of depression once per year. The change in the depression sum score from pre- to post-treatment time points is to be analyzed.

### Quality of life assessment

Brain cancer severely affects patients’ QoL, which is to a great extent caused by cognitive dysfunction and personality changes due to treatment or the disease itself [4, 41, 42]. Here it is hypothesized that patients’ health-related QoL (HRQoL) will decrease less in the HA-WBRT+SIB group than in the WBRT+SIB group.

HRQoL is assessed by the European Organisation for Research and Treatment of Cancer (EORTC) Quality of Life Core Questionnaire for Palliative Care (QLQ-C15-PAL) [43], as well as the brain cancer-specific module QLQ-BN20 [44], which addresses symptoms that are specific to brain cancer and its treatment [45]. The QLQ-C15-PAL includes those domains and symptoms of the parent instrument, QLQ-C30 [46], identified as most relevant and important for palliative care, namely physical (two items) and emotional functioning (two items), the symptom scales fatigue (two items), and pain (two items), as well as five single items (dyspnea, insomnia, appetite loss, nausea, and constipation), and the global quality-of-life index [43]. This abbreviated questionnaire has shown better compliance and practicability compared to the QLQ-C30 [42]. The additional brain cancer-specific module QLQ-BN20 comprises eleven scales, among them four multi-item scales addressing future uncertainty (four items), visual disorder (three items), motor dysfunction (three items), and communication deficits (three items). The additional seven single-item scales assess headaches, seizures, drowsiness, hair loss, itchy skin, weakness of legs, and bladder control. The construct validity of the QLQ-BN20 has been tested and confirmed with regards to the EORTC QLQ-C30 [44] and it has shown a good reliability, with acceptable responsiveness to changes over time [45].

Symptom control and physical functioning is only one dimension of QoL and patients have their own coping resources to deal with their disease and therapy side effects. One of the best established instruments to evaluate Individual QoL (IQoL) is the Schedule for the Evaluation of Individual Quality of Life – Direct Weighting (SEIQoL-DW), an abbreviated form of the SEIQoL [47]. This highly valuable instrument asks about the satisfaction of the patients with different domains of life (e.g. family, work or health) and weights the results considering the individual relative importance of these domains for the patient. The SEIQoL-DW has been proven to be a valid and reliable measure of IQoL also in terminally ill patients. In our study the nomothetic measurement of HRQoL is complemented by the SEIQoL-Questionnaire (SEIQoL-Q) [48], developed to transform the interview guide of the original SEIQoL-DW into an economic written self-report that can be easily implemented in a high-volume radiotherapy unit.

The SEIQoL-Q is applied parallel to QLQ-C15-PAL and -BN20 before treatment and 3, 9, and 18 months after treatment. Beyond 18 months after the end of WBRT, all patients who are still alive receive HRQoL assessment with the SEIQoL-Q questionnaire once per year.

### Magnetic resonance imaging (MRI)

In this trial we also aim to investigate the putative benefit of HA-WBRT+SIB with state-of-the-art MRI methods. It is assumed that differential treatment effects will be detectable mainly within the hippocampus, in terms of volume change, reflecting effects on NCF. Therefore, hippocampal volume is measured at baseline and at follow-ups, in order to evaluate and compare changes between treatment groups. Similarly, brain volume changes after treatment are compared in order to detect brain atrophy.

Moreover, it is hypothesized that white matter toxicity (leukoencephalopathy) will be detectable initially in both treatment groups. We further hypothesize that patients receiving HA-WBRT+SIB will show a better recovery of lesions compared to patients receiving treatment without HA. It is assumed that this effect will arise from the sparing of the neural progenitor cells in the subgranular zone of the dentate gyrus of the hippocampus during HA-WBRT+SIB. Sparing of neural progenitor cells might hence allow for a better regeneration in terms of gliogenesis of white matter oligodendroglial cell populations.

Diffusion Tensor Imaging (DTI) studies in neurodegenerative diseases suggested that changes to the hippocampus are also associated with structural changes of the hippocampus output fibres. In patients with Alzheimer’s disease and Lewy Body Dementia, changes of the hippocampus correlated with decreased fractional anisotropy in the fornix and stria terminalis [49]. Decreased fractional anisotropy within outgoing fibres was discussed to be a result of Wallerian degeneration, with primary degeneration of hippocampal neurons leading to loss of output fibres. In another study increased diffusivity parameters were found in parahippocampal gyrus, posterior cingulum, adjacent temporo-parietal regions, splenium and fornix of patients with Alzheimer’s disease compared to age-matched healthy controls [50]. In children treated for acute lymphoblastic leukemia, DTI has previously been used to characterize neurotoxicity [51], with reduced fractional anisotropy after irradiation being associated with impaired neuropsychological test performance. Consequently, an extensive study-specific MRI protocol is planned for all follow-ups, including the following sequences: 3D fluid-attenuated inversion recovery (FLAIR), diffusion-weighted MRI (DWI), DTI, diffusion kurtosis imaging (DKI), T2 and T2* relaxometry, and a 3D T1-weighted, gradient-echo sequence (MP RAGE) before and after contrast agent injection.

### Local tumor control (LTC) and occurrence of new cerebral metastases

In order to ensure comparable efficacy of HA-WBRT+SIB and conventional WBRT+SIB, LTC and the occurrence of new cerebral metastases will be continuously monitored, especially within the hippocampal area. Study-specific MRI will be performed at baseline and 6 weeks, 3, 9, and 18 months after treatment. Clinical routine MRI will be performed 6 and 12 months after treatment. Beyond 18 months after the end of radiotherapy, documentation of progression based on clinical routine MRI is performed once per year for all patients who are still alive.

Time to progression is defined as the number of days from randomization to the first occurrence of the respective event. The date of occurrence is the first date on which progressive disease was suspected, if subsequently confirmed according to the study procedures.

Intracranial progression is defined as the occurrence of progressive disease (at least 20% increase in the sum of the longest diameter of the contrast enhancing lesion) concerning pre-existing brain metastases or the occurrence of new brain metastases [52]. Tumor progression in the area of dose escalation has to be distinguished from treatment-related changes. Progressive lesions can initially be treated conservatively for 6 weeks with dexamethasone (e.g. 3 × 4 mg/d for 4 days followed by a 2-mg dose reduction every 2 days) and pentoxifylline (400 mg twice a day). After 6 weeks, a control MRI scan is to be performed. If no further progression is noted, treatment-related changes are diagnosed and follow-up is continued. If a further increase in the contrast-enhancing lesions is seen, an intervention is initiated after interdisciplinary discussion, including surgery or biopsy – if medically and ethically indicated. Histological evidence of necrosis without vital tumor cells in a biopsy or resection leads to the diagnosis of radiation necrosis, whereas histological evidence of tumor or the initiation of further therapy leads to the diagnosis of local tumor progression.

Progression in the WBRT area without dose escalation is defined as occurrence of intracranial tumor progression (progression of existing lesions and/or occurrence of new lesions) in the WBRT area without dose escalation. Hippocampal progression is defined as occurrence of intracranial progression within the hippocampal avoidance region (HAR, hippocampus + 7 mm). For patients not diagnosed with the respective event at the end of the study, the respective time to progression will be censored at the time of the last available imaging assessment. Death before the occurrence of the respective progression is considered a competing event.

### Progression-free survival (PFS), overall survival (OS) and death caused by cerebral metastases

PFS will be calculated from randomization until the first occurrence of intracranial or extracranial progression, death without prior progression, or end of follow-up. For patients alive and not diagnosed with progression at the end of the study, the PFS time will be censored at the time the patient was last known to be free of intracranial progression based on imaging assessment and free of extracranial progression. Local PFS is defined as the number of days from randomization until local tumor progression, death without prior local progression, or end of follow-up. Patients alive and not diagnosed with local progression at the end of the study will be censored at the time of the last available imaging assessment.

OS will be calculated from randomization until death, and time to death caused by cerebral metastases will be calculated from randomization to death if caused by cerebral metastases. For patients alive the end of the study, OS will be censored at the time of the last follow-up. For the time to death caused by cerebral metastases, death from other causes is considered a competing event. Death due to cerebral metastases is defined as intracranial failure as a component of cause of death [53].

The time to extracranial progression will also be calculated from randomization until extracranial progression or end of follow-up, where extracranial progression is the first date on which progressive disease outside the brain occurred. For patients alive and not diagnosed with extracranial progression at the end of the study, the time to extracranial progression will be censored at the time the patient was last known to be free of extracranial progression. Death before the occurrence of extracranial progression is considered a competing event.

The application of systemic therapies prior or subsequent to irradiation is to be thoroughly documented throughout the study.

### Toxicity

Pre-defined adverse events will be reported with severity grades according to CTCAE. The radiotherapy applied in this trial could be associated with the following expected acute and sub-acute events: skin toxicity, alopecia, conjunctivitis, otitis, headache, vomiting, nausea, vertigo, fatigue, increase of lesion edema with consequent neurologic symptoms (e.g. aphasia, hemiparesis, cranial nerves disorders, cerebellar disorders, epileptic disorders), disturbances of wound healing after surgery. Systemic therapies (chemotherapy, immunotherapy, targeted therapies) are paused 1 week prior irradiation in order to minimize cumulative toxicity. An independent data safety monitoring committee, consisting of 2 physicians, a physicist and a statistician, is to be provided with all necessary information to review the specified adverse events by means of annual line listings.

### Biological specimens/pharmacogenetic studies (Medical center – University of Freiburg)

During clinical routine blood withdrawing, 20 ml Ethylenediaminetetraacetic acid blood and 8 ml blood in a Blue Tiger Top tube will be collected in the Medical Center – University of Freiburg only, at 4 distinct time points (before start of WBRT, after the last fraction, 6 weeks and 3 months after WBRT) after obtaining separate informed consent. Blood samples will be used to analyze circulating immune biomarkers as predictors of treatment response.

### Statistical planning and analysis

The sample size calculation is based on the primary outcome, which is defined as the difference between the learning scale of the VLMT (word count, 0–75 words) in survivors at 3 months after radiation therapy and at baseline.

Assuming a mean decline after WBRT+SIB of 15 words in VLMT and a common standard deviation of 15 words [6], the study is planned to reject the null-hypothesis of equality of treatment arms with 80% power on the basis of a two-sample t-test at a one-sided significance level of 10% if the deterioration of NCF improves to a 7 words decline after HA-WBRT+SIB. Under these assumptions, 66 patients are required for analysis. According to the patients’ prognosis, we expect about 55% of patients in both arms to survive until 3 months after radiation, plus some additional drop-out of patients unwilling or unable to perform the test. We reckon that 50% of randomized patients will be available for analysis. Therefore, 132 patients should be randomized.

In the primary analysis, differences between HA-WBRT+SIB and WBRT+SIB with respect to the primary endpoint will be estimated and tested within a linear regression model in patients who survive until 3 months after radiation therapy. The model will include treatment and study center as independent variables and will adjust for the baseline learning scale word count of the VLMT. In case of endpoint values missing for reasons other than patient death, these will be handled by multiple imputation. The primary analysis population is the per-protocol set, defined as the group of randomized patients who had no major violations of eligibility criteria and who received treatment as per protocol according to their randomized arm. Sensitivity analyses of the primary endpoint as pre-defined in the study protocol will be performed.

Descriptive and explorative statistical analyses will be performed with respect to the secondary efficacy endpoints. Overall, PFS rates in the two treatment arms will be estimated by the Kaplan-Meier method, and the corresponding endpoints will be analyzed with the Cox model.

For safety analyses, rates of adverse events and the use of corticosteroids will be calculated with 95% confidence intervals.

## Discussion

The optimal treatment of multiple brain metastases has been the subject of increased controversy in the last decade and is a research question of great epidemiologic impact. While technical advances now allow the synchronous radiosurgical treatment of as many as 10 brain metastases [54, 55], insuring intracranial distant tumor control still remains imperative. Thus, despite known long-term side effects, WBRT is still the standard therapy for multiple (≥4) brain metastases [2, 3].

Approaches to improve the risk-benefit ratio for WBRT have been manifold. One of them was the sequential application of radiosurgeries to the metastases, used to increase the otherwise modest LTC [26]. In this case, the choice of a simultaneous boost application in the HIPPORAD-trial has the biological advantage of fractionation and ensures a better sparing of the hippocampi [27]. The tumor control and survival achieved in this trial will be compared with published data on WBRT alone and WBRT with sequential radiosurgeries to the metastases [24, 26, 53].

Another approach to preserve the integrity of cognitive functions involved the prescription of lower doses to the whole brain. While these appear to limit long-term adverse events such as atrophy and leukoencephalopathy, very low doses or no WBRT at all have also proved ineffective in insuring intracranial tumor control [3, 56]. The dose chosen in our trial for the CTV_WB_ (30 Gy/2.5 Gy) is only moderately lower than the standard 30 Gy in 10 fractions (with an equivalent dose in 2 Gy fractions, EQD2, α/ß = 10 of 32.5 Gy, a biological effective dose, BED, of 39 Gy and an EQD2 α/ß = 2 of 37.5 Gy). The dose applied in the HIPPORAD trial corresponds to an EQD2 α/ß = 10 of 31.25 Gy, BED 37.5 Gy and an EQD2 α/ß = 2 of 33.75 Gy. This slight de-escalation takes into consideration the smaller prescription volume (PTV_WB_ – PTV_met_ – HAR) and the allowed multiple boosts. Whether this approach will prevent whole brain atrophy and cognitive side effects, but come with the trade-off of poorer distant tumor control, as seen previously [22], has to be prospectively analyzed in this trial.

Recent data from the NRG Oncology CC001 study suggested that cognitive failure can be effectively prevented by conformal sparing of the hippocampi [19]. In comparison to this trial, HIPPORAD investigates not only HA-WBRT, but also the addition of a simultaneous dose escalation on the metastases. Not only high radiation doses, but also progressive intracranial tumor load can decrease cognitive function, as shown previously [23, 25]. Patients treated with HA-WBRT+SIB and WBRT+SIB could therefore experience less cognitive decline compared to both HA-WBRT and WBRT alone, as compared with reported data in the literature [6, 19]. Whether the sparing of the hippocampal stem cell niche and thus the preservation of local neurogenesis can help prevent overall cerebral atrophy has still to be evaluated. Morphological assessments are planned in the HIPPORAD trial, including overall volumetric measurements.

The multi-institutional prospective trial of Yamamoto et al. showed that radiosurgery alone can achieve comparable OS and cognitive toxicity rates, independent of the number of treated metastases within a range of 2–10 [57, 58]. However, in the first year after treatment, new lesions were reported in up to 64% of cases [58], reinforcing the idea that rdiosurgery carries the risk of failure in non-treated brain regions [59]. The HIPPORAD-trial takes into consideration the possible microscopic tumor spread and thus considers the whole brain as a CTV to be treated with 30 Gy in 12 fractions, with the goal of reducing intracranial relapses and neurologic death rates, as seen previously [53]. Furthermore, the dose delivered to the brain lesions is comparable to the one achieved by SRS, with an EQD2 α/ß = 10 of 60.6 Gy and a BED of 72.7 Gy.

The therapy concept in the HIPPORAD trial also takes into consideration the adjuvant radiotherapy of resection cavities with 42 Gy in 12 fractions. Local irradiation of the tumor bed was shown to significantly reduce the risk of local recurrence [60, 61], with postoperative stereotactic fractionated radiotherapy appearing to be more efficient compared to radiosurgery [60].

Concerning HA, the set dose constraints (D98% ≤ 9 Gy and D2% ≤ 17Gy) lead to a mean dose in the bilateral hippocampus of under 10 Gy (EQD2 α/ß = 2 of 7.1 Gy). These values are consistent with the constraints of the RTOG 0933 [18] and its subsequent NRG Oncology CC001 phase III trial [19], as well as with the dosimetric analyses of Gondi et al. [17], which revealed a limiting dose in 40% of the bilateral hippocampi (D40%) of 7.3 Gy for neurocognitive deterioration. In view of the reported biphasic development of cognitive dysfunction over time [62], the time points of NCF testing are considered appropriate for distinguishing acute, reversible decline from permanent cognitive failure. Furthermore, the planned tests aim to interpret cognitive changes within the context of life-altering health-related events, taking anxiety, depression and QoL into consideration.

In conclusion, we consider that the results of the multi-center prospective randomized HIPPORAD trial could significantly change the treatment strategy for multiple brain metastases, providing systematic and detailed information on a recently established method of therapy, which aims to achieve balance between tumor control and preserved QoL.

## Data Availability

Not applicable.

## Abbreviations

NCF: Neurocognitive function
HA: Hippocampus avoidance
SIB: Simultaneous integrated boost
WBRT: Whole brain radiation therapy
QoL: Quality of life
LTC: Local tumor control
PFS: Progression-free survival
OS: Overall survival
MRI: Magnetic resonance imaging
HAR: Hippocampal avoidance region
VLMT: German version of the Auditory Verbal Learning Test – “Verbaler Lern- und Merkfähigkeitstest”
SFRT: Stereotactic fractionated radiotherapy
OAR: Organ at risk
CT: Computed tomography
Gd-T1-MRI: T1-weighted sequence with intravenous Gadolinium-containing contrast medium of 1 mm isotropic voxel size
GTV: Gross tumor volume
CTV: Clinical target volume
PTV: Planning target volume
3D: Three-dimensional
D_98%_: Dose in 98% of the volume
D_2%_: Dose in 2% of the volume
WMS-R: Wechsler Memory Scale-Revised
TMT: Trail Making Test
HADS-D: Hospital Anxiety and Depression Scale
HRQoL: Health-related quality of life
EORTC: European Organisation for Research and Treatment of Cancer
QLQ-C15-PAL: Quality of Life Core Questionnaire for Palliative Care
QLQ-BN20: Brain cancer-specific module of the Quality of Life Core Questionnaire for Palliative Care
QLQ-C30: Quality of Life Core Questionnaire C30
IQoL: Individual QoL
SEIQoL: Schedule for the Evaluation of Individual Quality of Life
SEIQoL-DW: Schedule for the Evaluation of Individual Quality of Life - Direct Weighting
SEIQoL-DW-Q: Schedule for the Evaluation of Individual Quality of Life - Questionnaire
DTI: Diffusion Tensor Imaging
CTCAE: Common Terminology Criteria for Adverse Events
